# Brain Factor-7® improves learning and memory deficits and attenuates ischemic brain damage by reduction of ROS generation in stroke in vivo and in vitro

**DOI:** 10.1186/s42826-020-00057-x

**Published:** 2020-07-29

**Authors:** Yoohun Noh, Ji Hyeon Ahn, Ji-Won Lee, Junkee Hong, Tae-Kyeong Lee, Bora Kim, Sung-Su Kim, Moo-Ho Won

**Affiliations:** 1 Department of Anatomy and Cell Biology and Neurology, College of Medicine, Chung-Ang University, Seoul, 06974 Republic of Korea; 2 Famenity Co., Ltd., Uiwang, Gyeonggi 16006 Republic of Korea; 3 Department of Biomedical Science and Research Institute of Bioscience and Biotechnology, Hallym University, Chuncheon, Gangwon 24252 Republic of Korea; 4 Department of Neurobiology, School of Medicine, Kangwon National University, 1 Kangwondaehak-gil, Chuncheon, Gangwon 24341 Republic of Korea

**Keywords:** BF-7, Silk fibroin peptide, SH-SY5Y, Cerebral ischemia, 8-arm maze test, Passive avoidance test

## Abstract

Brain Factor-7® (BF-7), silk fibroin peptide, is known to be effective in improvement of memory and learning ability. In this study, the effects of BF-7 (10 mg/kg, p.o., pre-treatment for 7 days and post-treatment for 7 days) on neuroprotection and memory and learning functions were investigated in a rat model of transient focal cerebral ischemia and a gerbil model of transient global forebrain ischemia. Furthermore, to find the mechanism of BF-7, we examined the neuroprotective and antioxidative effects of BF-7 in vitro using neuroblastoma (SH-SY5Y) cells. In vivo model, treatment with BF-7 significantly reduced the number of errors in 8-arm maze test and significantly increased latency time in passive avoidance test at 7 days after focal ischemia compared to those in the vehicle-treated group. In addition, treatment with BF-7 significantly decreased the infarct size or neuronal death at 7 day following transient ischemia compared to that in the vehicle-treated group. In vitro model, 10 or 20 μg/ml of BF-7 treatment significantly increased cell viability in dose-dependent manner. In addition, oxidative stress was significantly attenuated in the ischemic cells, showing that 10 or 20 μg/ml of BF-7 treatment significantly reduced the generation of reactive oxygen species (ROS) compared to that in the ischemic cells. These results indicate that BF-7 treatment can attenuate ischemic damages and improve memory deficits via reduction of ROS generation.

## Introduction

Cerebral ischemia is closely related to the development of cognitive impairment [1]. The prevalence of cognitive impairment after stroke range is from 20 to 80% [2], more than a third of patients experience cognitive impairment after the transient cerebral ischemic attack [3]. It is known that a reduction of cerebral blood supply is closely related to brain dysfunction such as learning and memory impairment [4, 5] due to a reduction in the amount of acetylcholine in the brain [6], and contributes to the long-term disorder [7].

Ischemic stroke induced by cerebral ischemia is a common disease due to occlusion of cerebral blood flow supply. Cerebral ischemia causes cellular changes which are affected by free radical production, excitotoxicity, metabolic disorder, bioenergetics failure, and activation of proteases [8–10]. In addition, cerebral ischemic damage is accompanied by vascular leakage and inflammation, leading to apoptosis and tissue damage [9, 11]. By many recent studies, pathophysiological phenomenon or mechanisms of neuronal damage in ischemic brains have been investigated to prevent and protect the progression or consequences of cerebral ischemia. Therefore, many researchers are actively attempting a variety of approaches from various angles to develop new therapeutic agents [12, 13].

Three methods are widely known to treat cerebral ischemia to date: 1) rapid restoration of oxygen supply, 2) prevention of inflammation in ischemic area, and 3) protection of neural cells [14–16]. Current drugs in use or under development are antithrombic agents, immune and inflammatory modulators, free radical scavengers, Ca^2+^ channel antagonists, and NMDA/AMPA antagonists [17, 18]. However, these drugs remain highly controversial because of transient and weak effects, besides due to their serious side effects and toxicity. Thus, we need to find new therapeutic agents, which must have fewer side effects, to protect against ischemic injuries and to improve ischemia-induced brain dysfunctions.

It has been reported that Brain Factor-7® (BF-7), silk peptide, significantly reduces amyloid β-induced apoptosis in cultured human neuronal cell SKN-SH and increases cognitive function in healthy subjects [19, 20]. However, little has been investigated whether BF-7 protects against ischemic brain damage. Therefore, in the present study, we investigated the effects of BF-7 on learning and memory function and neuroprotection in animal models of cerebral focal or global ischemia. In addition, to examine the mechanism of the protective effect of BF-7, neuronal cell viability and the degree of reactive oxygen species generation were evaluated in vitro by using SH-SY5Y cells, which are neuroblastoma.

## Materials and methods

### Experimental animals

Twenty-one male Sprague-Dawley rats (8-week-old, 250–300 g of body weight) and twenty-one male Mongolian gerbils (6-month-old, 80–90 g of body weight) were obtained from the Experimental Animal Center, Kangwon National University (Chuncheon, Gangwon, Republic of Korea). The animals were housed at conventional states which were under adequate room temperature (24 ± 1 °C) and relative humidity (50 ± 5%). A constant cycle of dark/light was kept in every 12 h and freely accessible food pellets and water were provided to the animals ad libitum. The protocol applied in the present study was allowed by Institutional Animal Care and Use Committee (IACUC) at Kangwon National University (approval No., KW-200113-1) and adhered to the guidelines from the current international laws and policies in “Guide for the Care and Use of Laboratory Animals” (The National Academies Press, 8th Ed., 2011).

### Preparation of BF-7

BF-7 is a patented fibroin extract product manufactured by solubilization, degumming, desalination, enzymatic hydrolysis, and freeze-drying process from the silk (*Bombyx mori*) cocoon shell and it was supplied by Famenity Co., Ltd. [19, 21]. It has been reported that BF-7 is extracted form *Bombyx mori* and significantly reduces amyloid β-induced apoptosis in cultured human neuronal cell SKN-SH and increases cognitive function in healthy subjects [19, 21]. Since the post-treatment of 10 mg/kg BF-7 showed neuroprotective effect in the middle cerebral artery occlusion (MCAO) model [21], 10 mg/kg of BF-7 was selected in the present study.

### Experimental groups and BF-7 treatment

To examine whether BF-7 treatment attenuated ischemic brain damage (infarction) following focal cerebral ischemia, rats (*n* = 21) were randomly divided into three groups: (1) the sham-operated group (sham group), (2) vehicle treated ischemia-operated group (vehicle-ischemia group), (3) the BF-7 (10 mg/kg) treated ischemia-operated group (10 mg/kg BF-7-ischemia group). BF-7 or normal saline (vehicle) were orally administered once a day for 7 days before and for 7 days after inducing cerebral ischemia.

In addition, to examine neuroprotection against selective neuronal death induced by transient forebrain ischemia, gerbils (*n* = 21) were grouped as follow: (1) the sham-operated group (sham group), (2) vehicle treated ischemia-operated group (vehicle-ischemia group), (3) the BF-7 (10 mg/kg) treated ischemia-operated group (10 mg/kg BF-7-ischemia group). BF-7 or sterilized normal saline were orally administered once a day for 7 days before and for 7 days after inducing cerebral ischemia.

### Induction of MCAO in rats

The rats were initially anesthetized with a mixture of 2.5% isoflurane (Baxtor, Deerfield, IL, USA) in 33% oxygen and 67% nitrous oxide via face mask. Anesthesia was maintained with 2% isoflurane. A rectal temperature probe was introduced, and a heating pad maintained the body temperature at 37 °C during the surgery. Focal cerebral ischemia was induced by MCAO on the right side as described previously [22]. In brief, a 1.5 cm of midline incision was made onto the ventral neck of the animal and the sternocleidomastoid muscle was retracted laterally to expose the carotid artery, the internal carotid artery and the external carotid artery, and the arteries were split from adjacent tissues. Electrocautery was first applied to the superior thyroid artery branched from external carotid artery, larynx and arteries, and then to the pterygopalatine branched from the internal carotid artery. After the external carotid artery was dissected and 4–0 nylon silk was inserted to the internal carotid artery via the external carotid artery, the nylon was place in the 16–18 mm from the carotid bifurcation. The incision was closed, and nylon silk was removed an hour after the recovery from anesthesia. The animals which were given sham operation were undergone same surgical procedure without insertion of nylon silk.

### 8-arm radial maze test

According to our published procedure [11], spatial learning ability were examined in the rats using 8-arm radial maze test at 30 min after BF-7 treatment at 3 days to 7 days after MCAO following 5 days. The animals put in the maze for thirty minutes three times a day for 3 days before MCAO to allow adaptation. Briefly, 8-arm radial maze stands 4 cm from the floor consisting of eight equidistantly spaced arms (60 cm long and 12 cm wide), which are all radiating from an octagonal central platform with a radius of 34 cm radius and 40 cm wall. At the end of each arm, there is a food cup, and sunflower seeds were given as a reward. The light was dimmed, and rats were monitored with a video camera under a lamp (50 W). For training the rats, food supply was reduced to 80% from a week before the experiment. The number of visits and time to each arm has taken until all sunflower seeds were eaten, and rats were trained until the number of errors was less than two within two minutes. The number of errors was defined as the number of visits to the arms where rats had already visited. Then, the numbers of error and latency time were monitored for the following 5 days and compared with the control group.

### Passive avoidance test

According to our published procedure [23], short-term memory functions were examined using passive avoidance test at 30 min after BF-7 treatment at 6 days and 7 days after MCAO. As an experimental instrument, an automated shuttle box (Model PACS-30, Coumbus Instruments International Company, OH, USA) was used. The shuttle box is divided into two rooms of the same size by the partition door, and the floor is equipped with an electric current. Each room can be illuminated with a 20 W light bulb placed on a hinged plexiglass lid. The noise was less than 60 dB, and the experiment was conducted in a dark room. For training, the rat is first placed in a room with a light on, the partition door is open, the rat examines the room, and then the rat moves to a dark room. At this time, the partition door is automatically closed, and the light is turned off. Repeated this training 5 times until the rat enters the darkroom within twenty seconds. On the 6th trial, when the rat was placed in a lighted room and then entered the darkroom, the partition door closes, and a current (1 mA) flows for 3 s from the bottom of the shuttle box. After 24 h after completing the training process, put the rat back into the lighted room of the shuttle box, and measure the latency time until the rat goes to the darkroom. The maximum time to enter the darkroom is five minutes.

### Measurement of infarct volume

According to our published procedure [11], the affected areas of cerebral infarct were measured at 7 day after MCAO. Through intraperitoneal injection of pentobarbital sodium (60 mg/kg, JW Pharm Co Ltd., Republic of Korea), rats (*n* = 7 per group) were deeply anesthetized [24]. And, thereafter, they were decapitated, and the brain was removed. The whole brain was placed on the rodent brain matrix (RBM-4000C, ASI, USA), and coronal sections were made at a 2 mm interval from the end of the frontal lobe. The slices were incubated in 2% 2, 3, 5-triphenyltetrazolium chloride (TTC, Sigma-Aldrich, St. Louis, MO, USA) for thirty minutes and then fixed with 10% neutral buffer formalin.

After TTC reaction, infarct volume was measured using Image J analysis software (version 1.6 NIH). Unstained areas (pale color) were defined as ischemic lesions. The infarct volume was calculated according to the slice thickness of 2 mm per section. Each side of the brain slices was measured separately, and the mean values were calculated. Infarction area at each section is expressed as a percentage of corresponding brain section volume.

### Induction of transient forebrain ischemia in gerbils

The surgical procedure was performed as previously described [25]. In short, using an inhaler (Hana Pharmaceutical Co., Ltd., Seoul, Republic of Korea), the gerbils were undergone respiratory anesthesia with a mixed gas of 2.5% isoflurane (Baxtor, Deerfield, IL, USA) in 33% oxygen and 67% nitrous oxide. The ventral neck of the gerbils was shaved and made a 1 cm of midline incision. The bilateral common carotid artery (BCCA) was exposed and ligated with non-traumatic aneurysm clips for 15 min. Thereafter, the clips were removed in order to reperfusion. Using an ophthalmoscope (Heine K180®, Heine Optotechnik, Herrsching, Germany), complete occlusion and reperfusion of the BCCA were confirmed via observing blood flows in the central arteries of retinae. The body temperature was maintained in normothermic condition (36.8 ± 0.2 °C) and monitored with a rectal temperature probe (TR-100; Fine Science Tools, Inc., Foster City, CA, USA) during the surgical procedure. The sham operated gerbils were undergone the identical surgical procedure without ligation of BCCA.

### Observation of selective neuronal death

To investigate histopathological analysis, tissues were prepared in accordance with a previously described method [25]. Briefly, at 7 days after ischemic insult, the gerbils were anesthetized via intraperitoneal injection of pentobarbital sodium (70 mg/kg, JW Pharm Co Ltd., Republic of Korea) [24]. Next, they were transcardially perfused with 0.1 M phosphate-buffered saline (PBS, pH 7.4) followed by 4% paraformaldehyde (in 0.1 M phosphate buffer, pH 7.4). Their brains were harvested and post-fixed with the same fixative for 2 h at room temperature. To cryoprotect, the brains were infiltrated with 30% sucrose solution (in 0.1 M phosphate buffer, pH 7.4) for 24 h at room temperature. Finally, the brains were coronally sectioned into 10 μm thickness using a sliding microtome (Leica, Nussloch, Germany).

Cresyl violet (CV) staining was carried out according to a previously published method [25]. In short, the sections were mounted on microscopy slides coated with gelatin. The sections were subsequently stained with 1.0% (w/v) CV acetate solutions. Next, the stained sections were dehydrated via immersing into serial ethanol bath and mounted with Canada balsam (Kanto, Tokyo, Japan) and cover glasses.

Fluoro-Jade B (F-J B, a marker for degenerative neuron) with a highly affinitive fluorescent histofluorescence staining was conducted. As described previously [25], in brief, the sections were immersed into potassium permanganate solution (0.006% w/v, in distilled water) and incubated with a solution of 0.0004% F-J B (Histochem, Jefferson, AR, USA) (in 0.1% w/w glacial acetic acid). The sections were finally dehydrated and covered using dibutylphthalate polystyrene xylene (DPX) (Sigma, St. Louis, MO, USA).

To analyze changes in CV- and F-J B- positive cells, the images of CV staining and F-J B histofluorescence were obtained by using light microscope (BX53) (Olympus, Hamburg, Japan) and epifluorescent microscope (470–490 nm of blue excitation light) (Carl Zeiss, Oberkochen, Germany), respectively, which were equipped with a digital camera (DP72) (Olympus, Japan) coupled with a PC monitor. Cell count was done by averaging total numbers of the CV- and F-J B-positive cells using an image analyzing system (software: Optimas 6.5) (CyberMetrics, Scottsdale, AZ, USA).

### Cell preparation in vitro

SH-SY5Y (human neuroblastoma) cells were provided by the Korean Cell Bank (Seoul, Republic of Korea). Dulbecco’s modified Eagle’s medium and 10% heat-inactivated fetal bovine serum (FBS) were purchased from Thermo Fisher Scientific (MA, USA).

### Cell culture and cell viability assay

According to the previous study [26], Human neuroblastoma SH-SY5Y cells were cultured at 37 °C in Dulbecco’s modified Eagle’s medium (DMEM) supplemented with 10% FBS which was inactivated by heat in a humidified 95% air, 5% CO_2_ incubator. The cells were transferred to low serum media (10% FBS) for two hours before the treatment with BF-7.

The SH-SY5Y cells were plated on 96-well plates at a density of 5 × 10^4^ cells/well in 100 μl of 10% FBS/DMEM and incubated for twenty-four hours. The media was replaced with 90 μl of 10% FBS/DMEM. The BF-7 (10 and 20 μg/ml) were treated to cells and incubated for four hours. And then, H_2_O_2_ (0.25 μM) was treated to cells and incubated for twenty-four hours. After the treatment, cell viability was evaluated according to the previous study [23], in brief, 10 μg of 3-(4, 5-dimethylthiazol-2-yl)-2, 5-diphenyltetrazolium bromide (MTT) solution was aseptically added. The cells were incubated for three to four hours, and the absorbance of the cells was measured at a wavelength of 570 nm using ELISA Reader. The cell viability was defined as [(test sample count)-(blank count)/(untreated control count)-(blank count)] × 100 [27].

### Determination of reactive oxygen species (ROS) generation

Hydrogen peroxide generation induced by H_2_O_2_ was measured by incubation with a fluorescent probe 2′, 7′-dichlorofluorescein diacetate (DCF-DA), according to the previous study [23]. The cells (SH-SY5Y, neuroblastoma cell) were plated on 96-well plates at a density of 5 × 10^4^ cells/well in 200 μl of 10% FBS/DMEM and incubated for twenty-four hours. The media was replaced with 160 μl of 10% FBS/DMEM. The BF-7 (10 and 20 μg/ml) and DCF-DA solution were added to the cell and incubated for four hours. And then, H_2_O_2_ (0.25 μM) was added to the cell and incubated for two hours. The absorbance of the cells was measured with excitation at 485 nm and emission at 530 nm by a fluorometer.

### Statistical analysis

The data were presented as means ± standard error of the mean (SEM). Differences of the means among the experimental groups were statistically analyzed through Student’s t-test for infarction area, one-way ANOVA for latency time and ROS generation, and two-way ANOVA for number of errors. All statistical analysis was performed using SPSS 17.0 software (IBM, NY, USA) and *P* < 0.05 was considered as statistical significance.

## Results

### 8-arm maze test in rats

**Fig. 1:**
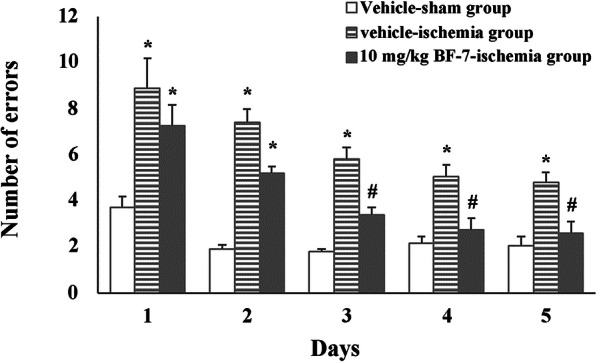
The beneficial effect of BF-7 on learning and memory impairment using an 8-arm maze test against focal cerebral ischemia. In the 10 mg/kg BF-7-ischemia group, numbers of errors are significantly reduced compared to those in the vehicle-ischemia group. The bars indicate mean ± SEM. *P < 0.05 compared with the vehicle-sham group; #P < 0.05 compared with the vehicle-ischemia group (*n* = 7)

The number of errors from 1 day to 5 days showed nearly no difference in the in the sham group (Fig. 1). In the vehicle-ischemia group, the number of errors was significantly higher compared to that in the vehicle-sham group (Fig. 1). In contrast, the number of errors in the 10 mg/kg BF-7-ischemia group, from the third day the number of errors showed significantly decreased compared to that in the vehicle-ischemia group (Fig. 1). Until the last day of 8-arm maze test, the number of errors in the 10 mg/kg BF-7-ischemia group decreased to the number of errors in the vehicle-sham group (Fig. 1). In contrast, the number of errors in the vehicle-ischemia group was not fully recovered (Fig. 1).

### Passive avoidance test in rats

**Fig. 2:**
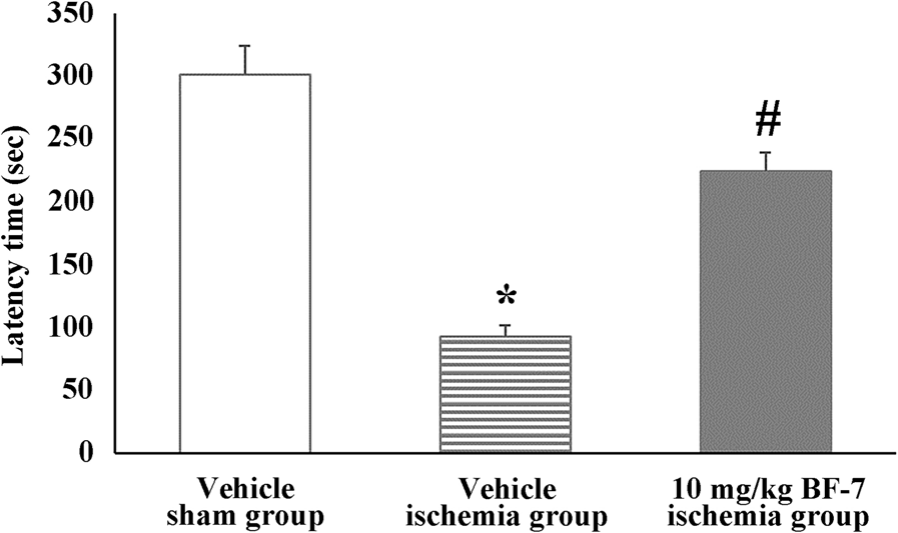
The beneficial effect of BF-7 on learning and memory impairment using passive avoidance test against focal cerebral ischemia. In the 10 mg/kg BF-7-ischemia group, latency time is significantly shortened compared to that in the vehicle-ischemia group. The bars indicate mean ± SEM. *P < 0.05 compared with the vehicle-sham group; #P < 0.05 compared with the vehicle-ischemia group (*n* = 7)

In the sham group, the animals were stayed in lighted room for 301.4 ± 22.3 s (Fig. 2). In the vehicle-ischemia group, the latency time was 92.7 ± 9.2 s, which was significantly reduced compared to that in the vehicle-sham group (Fig. 2). On the other hand, in the 10 mg/kg BF-7-ischemia group, the latency time was significantly increased (224.5 ± 14.9 s) compared to that in the vehicle-ischemia group (Fig. 2). Based on the above result, the spatial memory decreased due to MCAO and the decline in spatial memory was abolished by treatment with BF-7.

### Infarction in rats

**Fig. 3:**
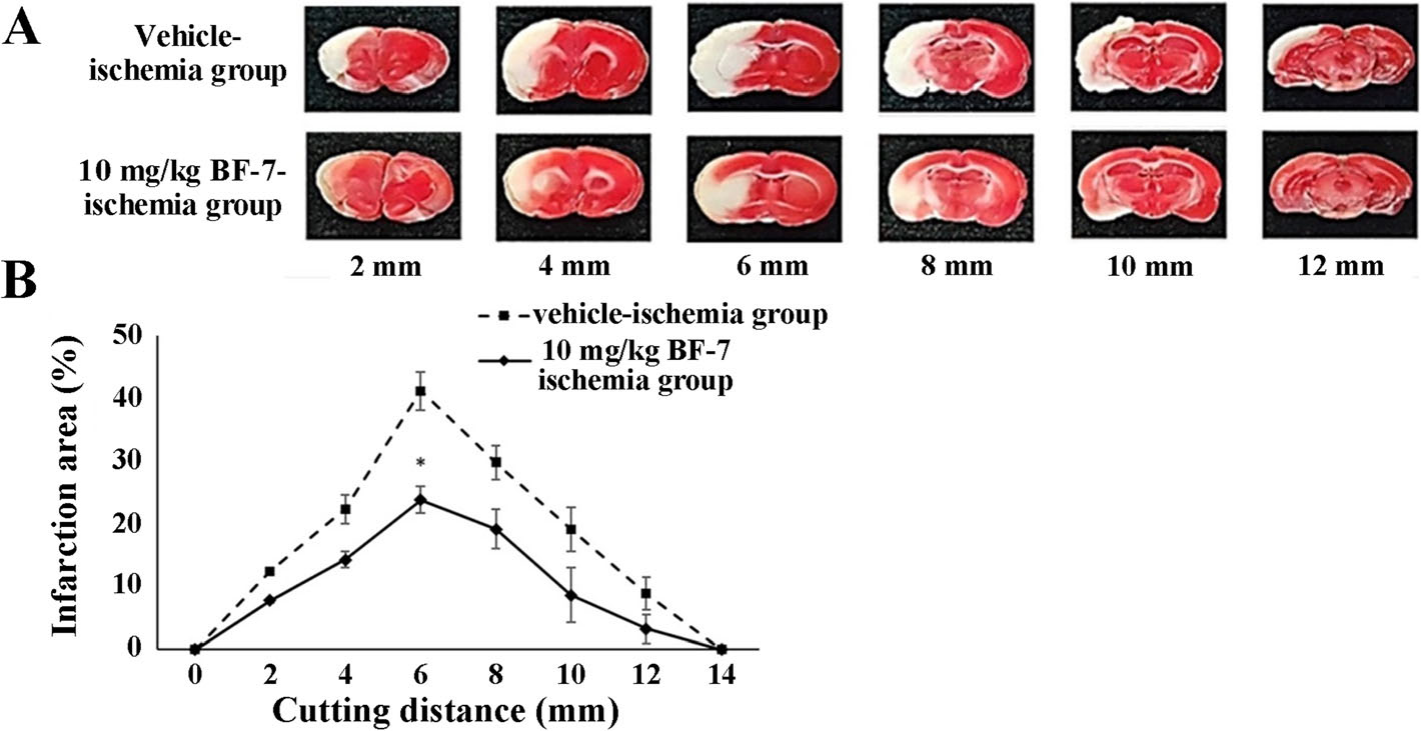
The effect of BF-7 on infarction area by MCAO. **a** TTC staining. The white area represented an infarcted lesion. **b** Size of infarcted area. In the 10 mg/kg BF-7 group, infarct areas are decreased compared to those in the vehicle-ischemia group. The bars indicate mean ± SEM. *P < 0.05 compared with vehicle-ischemia group (*n* = 7)

There no infarct lesion was observed in the vehicle-sham group (data not shown). In the vehicle-ischemia group, advanced infarct lesion was developed due to MCAO (Fig. 3a). In vehicle-ischemia group, especially, the largest infarct area was detected in the 6 mm portion form frontal lobe pole (Fig. 3a). However, in the 10 mg/kg BF-7-ischemia group, the infarction area tended to decrease compared to that in the vehicle-ischemia group (Fig. 3a, b). In particular, in the 6 mm portion from the frontal lobe pole, that is, the area where the damage is mostly caused by ischemic damage, a significant attenuation in the cerebral infarction site was found (Fig. 3a, b).

### Selective neuronal death in gerbils

**Fig. 4:**
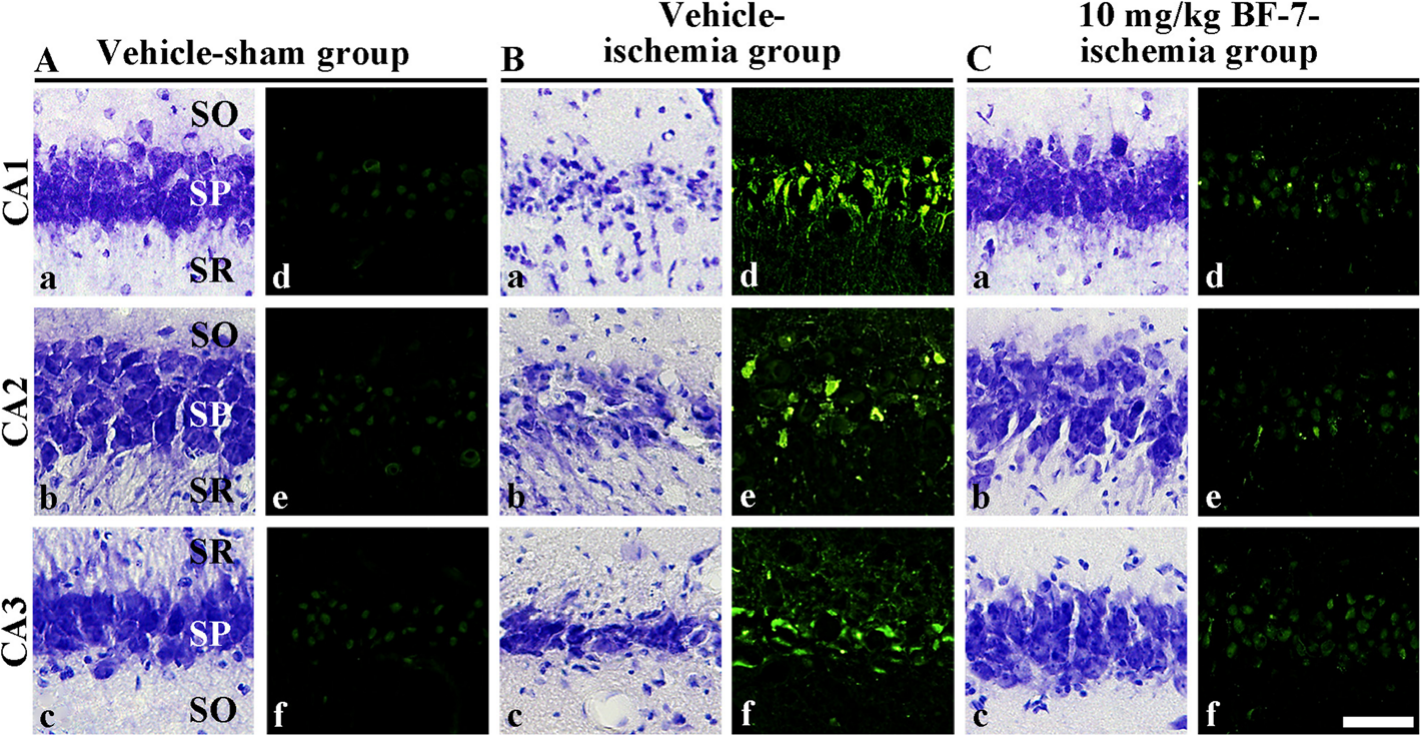
CV staining (**a**-**c**) and F-J B histofluorescence staining (**d**-**f**) in gerbil hippocampal CA1–3 regions of the vehicle-sham group (**a**), vehicle-ischemia group (**b**) and 10 mg/kg BF-7-ischemia group (**c**) at 7 days after transient forebrain ischemia. In the vehicle-ischemia group, pyramidal neurons in the CA1–3 regions show weak CV stainability and no F-J B histofluorescence. In contrast, in the 10 mg/kg BF-7-ischemia group, cells in the stratum pyramidale (SP) are well stained with CV, showing that a few F-J B positive cells are detected in the SP. SO, stratum oriens; SR, stratum radiatum Scale bar = 50 μm (*n* = 7)

In the vehicle-sham group, pyramidal neurons in all hippocampal subregions were well stained with CV (Fig. 4Aa-Ac). In this group, no F-J B positive cells were detected in the hippocampus (Fig. 4Ad-Af). However, in the vehicle-ischemia group, cells located in the stratum pyramidale in the CA1–3 regions of the hippocampus showed weakened CV dyeability and F-J B fluorescence (Fig. 4b). On the other hand, in the 10 mg/kg BF-7-ischemia group, distribute patterns of CV-stained cells were similar to those in the vehicle-sham group (Fig. 4Ca-Cc). In this group, a few F-J B positive cells were observed in in the stratum pyramidale of the CA1–3 regions of the hippocampus (Fig. 4Cd-Cf).

### Protective effects of BF-7 in vitro

**Fig. 5:**
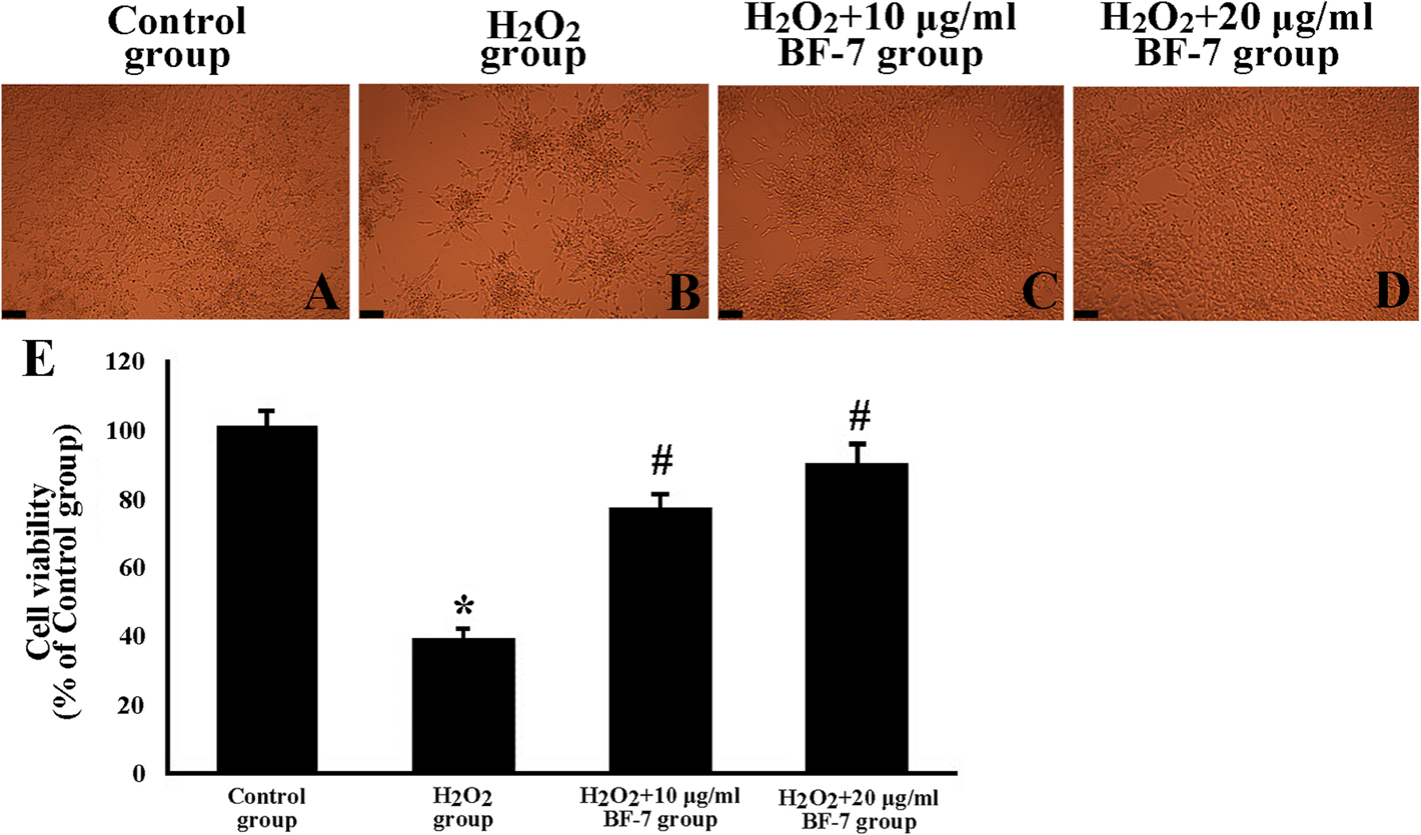
Effect of BF-7 on H_2_O_2_ induced cell death in SH-SY5Y cells of the control group (**a**), H_2_O_2_ group (**b**), and H_2_O_2_ + 10 μg/ml BF-7 (**c**) and H_2_O_2_ + 20 μg/ml BF-7 (**d**) groups. Morphological changes are detected in association with H_2_O_2_ toxicity. However, in the H_2_O_2_ + 20 μg/ml BF-7 group, the pattern of the cellular distribution is similar to that in the control group. E: cell viability (%), the control group is designated at 100%. Scale bar = 100 μm

In the control group, normal cellular distribution and morphology were observed (Fig. 5a). However, in the H_2_O_2_ group, condensation and segmentation of the cell bodies were found, and the typical apoptotic pattern was detected when the external change of cells was observed to investigate the morphological apoptotic pattern (Fig. 5b). On the other hand, in the H_2_O_2_ + 10 and 20 μg/ml BF-7 groups, it was confirmed that the degree of apoptosis was suppressed, and this was confirmed to suppress apoptosis in a dose-dependent manner (Fig. 5c and d).

### Production of ROS by H_2_O_2_ and its suppression by BF-7

**Fig. 6:**
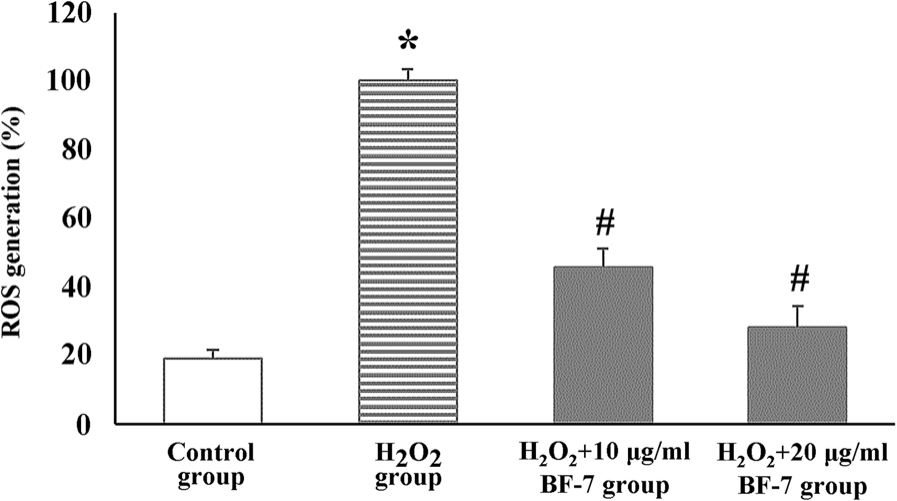
Effect of BF-7 on H_2_O_2_ induced ROS in SH-SY5Y cells. The bars indicate mean ± SEM. The ROS generation in the BF-7 treated groups is remarkably reduced compared to that in the control group. *P < 0.05 compared with the control group; #P < 0.05 compared with the H_2_O_2_ group

In the control group, general ROS generation (19.2 ± 2.4%) was found (Fig. 6). However, in the H_2_O_2_ group, the ROS generation was significantly increased (Fig. 6). In the H_2_O_2_ + 10 and 20 μg/ml BF-7 groups, On the other hand, significantly moderated ROS generations were detected in a dose-dependent manner compared to those in the in the H_2_O_2_ group (Fig. 6).

## Discussion

Until now, various kinds of natural resources have been studied because of their neuroprotective potentiality against cerebral ischemia. For example, a previous study showed that aqueous extract from *Scrophulariae Radix,* which belongs to Scrophulariaceae family, ameliorated infarct lesion in the brain of a rat model of MCAO via regulation of mitogen-activated protein kinase pathway [28]. In addition, we reported that laminarin and fucoidan, which are brown algae-derived polysaccharides, exerted neuroprotective effects against 5-min transient forebrain ischemia in gerbils through their antioxidant efficacy [29, 30]. To the best of our knowledge, even though various natural resources display neuroprotective effects, the neuroprotective effect of cocoon hydrate such as BF-7 against ischemic insults has been poorly investigated. In this study, therefore, to examine neuroprotective effects and attenuation of cognitive impairment by BF-7 following ischemic insults, we used well-established in vivo models for cerebral ischemia as follows: 1) transient focal cerebral ischemia induced by temporary blockage of the middle cerebral artery in rats, and 2) transient forebrain ischemia due to transitory occlusion of in gerbils BCCA. In addition, the mechanism of the protective effect of BF-7 related to antioxidative property was investigated using SH-SY5Y cells.

Many studies have demonstrated that decline in learning and memory function is closely related to hippocampus damage in in vivo cerebral ischemia models [6, 31]. In the present study, cognitive function was evaluated via passive avoidance test and 8-arm radial maze test following focal cerebral ischemia in rats. The results showed that the numbers of errors were significantly decreased in the 8-arm radial maze test, and the latency time in the passive avoidance test was significantly increased in the pre and post BF-7 treated groups compared to that in the vehicle-ischemia group. This finding was consistent with the previous studies that reported that BF-7 treatment significantly reduced the number of errors in 8-arm radial maze test after MCAO in rats [21], and BF-7 treatment significantly increased memory function in a dose-dependent manner in healthy humans (ages from 17 to 64) evaluated by Rey Complex Figure Test (RCFT) [19, 20], in which the method is common to evaluate memory and composition ability of the frontal lobe in brains responsible for memory, analysis, calculation, thinking, and judgment [32]. Therefore, the results of our and previous studies indicate that pre and post administration of BF-7 is effective in improving memory impairment following cerebral ischemia.

In our in vivo assessments, the infarct lesion was visualized by TTC assay following focal cerebral ischemia induced by MCAO in rats [28, 33], and the delayed neuronal death in the hippocampal CA1–3 regions was confirmed by CV staining and F-J B histofluorescence following 15-min of transient forebrain ischemia in gerbils [34–37]. In this study, compared with the vehicle-ischemia group, the infarct area in the BF-7-administered group was significantly reduced after MCAO in rats. In addition, pre and post BF-7 administration in the gerbil model of BCCA occlusion for 15 min significantly decreased the number of FJ-B cells (degenerating or dead cells) in the hippocampus compared with the vehicle-ischemia group. It was reported that BF-7 treatment after MCAO significantly decreased infarct volume in the rat brain [21]. To the best of our knowledge, neuroprotective effects in other cocoon hydrolysate were not investigated, but only in BF-7 [38]. In this study, we firstly demonstrated the neuroprotective effect of BF-7 against transient forebrain ischemia in gerbils. This result implies that BF-7 possesses neuroprotective potential against ischemic insults.

In our current in vitro assessments, cell viability was significantly decreased, and ROS generation was significantly increased when oxidative stress by H_2_O_2_ was applied to SH-SY5Y cells, however, BF-7 treatment exerted cellular protective effects and significantly inhibited ROS generation in dose-dependent manner. Similarly, previous studies showed that BF-7 significantly decreased ROS generation and resulted in the prevention of amyloid β (Aβ) peptide-induced apoptosis in human neuroblastoma SKN-SH cells [19] and FeSO_4_-induced apoptosis in neuroblastoma SK-N-SH cells [39]. In addition, silk fibroin hydrolysate significantly attenuated ROS accumulation and up-regulated superoxide dismutase (SOD) gene expressions, and resulted in the protection of Aβ_25–35_ induced cytotoxicity in primary hippocampal neurons [40]. Based on our current results and the results of the previous studies, it is likely that BF-7 can protect neural cells and that the protection is closely related to its antioxidant activity against oxidative damage.

## Conclusion

In summary, the results of our current study showed that BF-7 administration significantly attenuated cognitive deficits and prevented neuronal cell damage following focal and global cerebral ischemia in rodents. In addition, BF-7 treatment significantly reduced ROS generation in vitro. These results indicate that BF-7, a silk peptide, could protect neuronal cells from ischemic injury by inhibiting ROS generation. Therefore, it is considered to be of sufficient value to develop more accurate pharmacological mechanisms as a therapeutic agent for cognitive impairment caused by cerebral ischemia.

## Data Availability

All data produced and analyzed in the present study are included in this published paper.

## Abbreviations

Aβ: Amyloid β
BF-7: Brain Factor-7®
BCCA: Bilateral common carotid artery
CA: Cornu ammonis
CV: Cresyl violet
DCF-DA 2′: 7′-dichlorofluorescein diacetate
DMEM: Dulbecco’s modified Eagle’s medium
FBS: Fetal bovine serum
F-J B: Fluoro-Jade B
MCAO: Middle cerebral artery occlusion
MTT: 3-(4,5-dimethylthiazol-2-yl)-2,5-diphenyltetrazolium bromide
PBS: Phosphate-buffered saline
ROS: Reactive oxygen species
RCFT: Rey complex figure test
SOD: Superoxide dismutase
TTC: 2, 3, 5-triphenyltetrazolium chloride
